# Editorial: The Responses of Marine Microorganisms, Communities and Ecofunctions to Environmental Gradients

**DOI:** 10.3389/fmicb.2019.00115

**Published:** 2019-02-08

**Authors:** Hongyue Dang, Martin G. Klotz, Charles R. Lovell, Stefan M. Sievert

**Affiliations:** ^1^State Key Laboratory of Marine Environmental Science, Institute of Marine Microbes and Ecospheres, and College of Ocean and Earth Sciences, Xiamen University, Xiamen, China; ^2^School of Molecular Biosciences, Washington State University, Richland, WA, United States; ^3^Department of Biological Sciences, University of South Carolina, Columbia, SC, United States; ^4^Biology Department, Woods Hole Oceanographic Institution, Woods Hole, MA, United States

**Keywords:** marine microbiology, microbial ecology, biogeochemical cycles, environmental gradients, global change, ocean acidification, greenhouse gases

From estuaries to marginal seas and open oceans, from tropical warm pools to subtropical gyres and polar cryospheres, from sunlit surface water to twilight zone and pitch-black abyssopelagic water, from water columns to sediments and deep subseafloor biospheres, marine ecosystems experience diverse environmental gradients (Karl, 2007; Dang and Jiao, 2014). In addition to these large-scale gradients, small-scale, and micro-scale gradients of various physicochemical factors are common in the ocean; in particular, in marginal seas and coastal environments (Kappler et al., 2005; Stocker, 2012). The diverse gradients of physicochemical parameters, nutrients, and chemicals serving as electron donors and acceptors contribute to the creation of habitat heterogeneity and novel locales along a gradient may create unique niches for any given microorganism. Whether at the surface of a marine snow particle or alga, at the edges of an oxygen minimum zone (OMZ), in marginal sea methane-seep sediments, or on a chimney wall of a deep-sea hydrothermal vent, these interfaces provide hotspot habitats with sharp physicochemical gradients that may host diverse yet unknown microorganisms that facilitate yet unknown biogeochemical processes (Hügler and Sievert, 2011; Wright et al., 2012; Dang and Lovell, 2016). With the progress of marine molecular microbial ecology and “omics” techniques, certain environmental keystone microorganisms have been discovered at some of these interfaces: such as the anaerobic methane-oxidizing (ANME) archaea in methane-rich sediments (Valentine and Reeburgh, 2000), cable bacteria that facilitate electrogenic sedimentary sulfide oxidation (Nielsen and Risgaard-Petersen, 2015), neutrophilic zetaproteobacterial iron-oxidizing bacteria (FeOB) in deep-sea hydrothermal microbial mats and at abyssal basaltic glass-seawater and coastal metal-seawater interfaces (Emerson et al., 2010; Dang et al., 2011; Henri et al., 2016), anaerobic ammonium-oxidizing (anammox) bacteria and SUP05 sulfur-oxidizing bacteria (SOXB) in coastal and oceanic OMZs (Dick et al., 2013; Oshiki et al., 2016), and sulfur-oxidizing and/or hydrogen-oxidizing *Campylobacteria* in the proposed new phylum *Campylobacterota* (formerly known as *Epsilonproteobacteria*; Waite et al., 2018) at seawater, hydrothermal vent, and subseafloor redox interfaces (Campbell et al., 2006; Grote et al., 2012; Dick et al., 2013; Han and Perner, 2015; McNichol et al., 2018). Even the ubiquitous marine ammonia-oxidizing *Thaumarchaeota*, discovered only a decade ago (Könneke et al., 2005), can now be divided into two distinct ecological groups according to the vertical physicochemical profile of marine water, the “shallow clade” and the “deep clade” (Hatzenpichler, 2012). The ongoing discovery of unique ecophysiological functions of marine Bacteria and Archaea will contribute to a conceptual rewriting of biogeochemical pathways in the marine C, N, S, and Fe cycles.

The characterization of how the abundance and spatial distribution of marine microorganisms, the structure of microbial communities and their provided ecosystem functions respond to the diverse environmental gradients is of fundamental importance to our understanding of the microbial ecology and biogeochemistry of the oceans. This rationale defines the aim and scope of this Research Topic. The contributions of environmental gradients to the diversity of marine microorganisms and their metabolic potentials may play important roles in maintaining the stability and functions of the estuarine, coastal and marginal sea ecosystems, which have been experiencing a multitude of anthropogenic perturbations (Dang and Jiao, 2014; Damashek and Francis, 2018). The responses of the affected microbial communities to human-induced environmental impacts are currently still difficult to predict and the understanding of microbial processes and mechanisms at the community level is the key for predictive modeling, which also requires the collection of large empirical data sets (Haruta et al., 2013; Hanemaaijer et al., 2015; Burd et al., 2016). Greater understanding of microbial responses to natural and anthropogenic environmental gradients may also help us understand the responses of marine ecosystems to global climate change and other large-scale environmental perturbations such as ocean acidification and spatial and temporal ocean deoxygenation.

The authors of this Research Topic contributed a total of 21 publications covering a wide variety of subjects spanning from microbial metabolic dynamics to biogeochemical cycling of C, N, S, and Fe in micro-, small-, and geographic-scale marine gradients. This Editorial aims to highlight some of the main findings reported in these publications and we would like to take this opportunity to thank all participating editors and reviewers for making this Research Topic a success. In order to cover the broad subjects of the articles published in this Research Topic, we organize our introductions to these publications in the following sequence: (1) microbial ecoenergetic strategies and dynamics in response to energy sources and dynamics; (2) microbial community structure variations and their impacts on marine C, N, S, and Fe cycling in response to natural and anthropogenic gradients; and (3) microbial regulatory processes and mechanisms in response to environmental gradients and variations. We closed this Editorial by referring to the need for ongoing experimental advancement in future studies of marine microorganisms, their communities and ecofunctions, in response to marine environmental changes.

## Microbial Ecoenergetic Responses to Energy Sources and Dynamics

Sunlight is the major energy source that supports most of the primary production in surface oceans and in sediments under shallow water; hence, light availability controls the vertical distribution of photosynthetic communities in the ocean. However, the photochemical energy conversion efficiency of the phytoplankton communities was recently found to be relatively low and further limited by nutrient scarcity in vast regions of the ocean (Lin et al., 2016; Falkowski et al., 2017). Lichtenberg et al. investigated radiative energy budgets and energy conversion efficiency of benthic phototrophic microbial communities in coral reef sediments and a cyanobacterial biofilm. They found that local photosynthetic efficiencies change as function of physical structure of microbial communities and gradients of diffuse or collimated light further change the pattern of radiative energy conversion. In addition to light energy, energy stored in chemical bonds is also explored by microorganisms for carrying out dark inorganic carbon fixation (Hügler and Sievert, 2011), which may play an important role in marine carbon cycling and climate modulation. For example, in a very recent investigation in the South China Sea, the integrated water column dark carbon fixation rate was estimated to be nearly 4-fold of euphotic zone primary production (Zhou et al., 2017). The review article by Dang and Chen further discussed the eco-energetic strategies of key marine chemolithoautotrophic nitrogen-cycling microorganisms and their tentative responses to marine environmental changes such as those caused by global warming, ocean acidification, deoxygenation, eutrophication, and heavy metal pollutions.

## Microbial Community Responses to Natural and Anthropogenic Gradients and Their Impacts on Marine C, N, S and Fe Cycling

Cyanobacteria are key primary producers in the ocean. Mackey et al. revealed variations in the oligotypes of *Synechococcus* strains and thus their microdiversity, relative abundances and niche differentiation in response to changes in season, and salinity in a salt marsh estuary by employing a novel molecular approach called oligotyping. Similarly, Xia et al. verified niche partitioning among *Synechococcus* in the Pearl River estuary, a salt wedge estuary of the South China Sea. Chen et al. showed that the biogeography of dominant planktonic and benthic microeukaryotic communities (possibly including autotrophs, heterotrophs, and mixotrophs) may be influenced mainly by environmental and spatial factors, while that of the rare subcommunities may be influenced by more complex mechanisms in the coastal environment of Xiamen, China.

Marine *Roseobacter* clade (MRC) bacteria are abundant as free-living and particle-associated microorganisms; particularly, in coastal waters, and some of them can carry out aerobic anoxygenic photosynthesis (Dang and Lovell, 2002, 2016; Buchan et al., 2014). He et al. investigated the seasonal and spatial distribution of the bacterioplankton communities in highly anthropogenically impacted Qinhuangdao coastal waters and reported that the bacterial abundance had significant positive correlation with seawater total phosphorus content, potentially serving as a key monitoring parameter for anthropogenic impact in the studied aquatic area. These authors also observed an inverse correlation between the dominant Family II *Cyanobacteria* and *Alphaproteobacteria* (mainly affiliated with the MRC). It will be worthwhile to further investigate what the ecological mechanism or controlling environmental factors are, if any, that determine the distinct spatial distribution of *Cyanobacteria* and MRC bacteria.

Shallow-water coral reefs are among the most productive and most diverse symbiotic ecosystems in the oceans (Cunning and Baker, 2014; Blackall et al., 2015; Peixoto et al., 2017). The response of coral microbiomes to environmental disturbance is highly complex (McDevitt-Irwin et al., 2017). Long-term surveys are critical to our ability to differentiate changes in response to anthropogenic disturbances from natural dynamics of the coral microbiomes. The work by Yang et al. highlights the importance of long-term surveys for coral microbial communities in revealing compositional shifts and environmental correlations and reported that the dominant bacterial groups in coral *Stylophora pistillata* showed differential geographical preference, whereas the composition of the minor bacterial members in *S*. *pistillata* fluctuated over time.

Although Archaea have been recognized as an important and diverse group of microorganisms in the ocean, knowledge gaps concerning the ecological and biogeochemical roles of many archaeal lineages remain (Offre et al., 2013; Spang et al., 2017). Ling et al. investigated chemolithoautotrophic ammonia-oxidizing *Thaumarchaeota* communities, along with communities of ammonia-oxidizing *Betaproteobacteria*, that were associated with the seagrass *Thalassia hemprichii* in several coral reef ecosystems of the South China Sea. Liu et al. reported much more abundant heterotrophic MG-II *Euryarchaeota* than chemolithoautotrophic *Thaumarchaeota* throughout the water column of the northeastern South China Sea and strong water mixing was inferred to be the cause of this unusual distribution pattern of the marine archaea. Furthermore, Wang et al. found that MG-II *Euryarchaeota* likely produce a large proportion of GDGTs, potentially important in marine carbon cycling (Zhang et al., 2015) and in revising the interpretation of TEX_86_, a paleotemperature proxy stored in marine sediments.

The global nitrogen cycle has been experiencing tremendous anthropogenic disturbances (Rockström et al., 2009). Whether nitrogen loss through denitrification and anammox, and nitrogen gain through microbial N_2_ fixation are presently still in balance in the anthropogenically-impacted modern ocean has been vigorously debated (e.g., Zhou et al., 2016; Dang and Chen). New diazotrophs and N_2_-fixing environments have recently been identified, including coastal sediments that harbor diverse and abundant sulfate-reducing bacteria (SRB) that are active in nitrogen fixation (Bertics et al., 2013; Dang et al., 2013; Pedersen et al., 2018; Zhou et al., 2016). The work by Zhang et al. showed the diazotrophic potential of SRB in the rhizospheres of tropical mangroves, which usually constitute highly productive intertidal ecosystems but meanwhile lack sufficient nutrients. Another environmental issue related to the contemporary nitrogen cycle is that N_2_O has emerged as the top ozone-destructing greenhouse gas (Voss et al., 2013). Microbial reduction is likely the sole biological sink for N_2_O, and the key enzyme in this process, nitrous oxide reductase, is known for its low oxygen tolerance (Bonin et al., 1989; Körner and Zumft, 1989). Nevertheless, Sun et al. reported in this Research Topic that composition of the active N_2_O-consuming microbial assemblages varied with seawater N_2_O but not O_2_ concentration across the oxic/anoxic gradient of the Eastern Tropical South Pacific Ocean. This work also tentatively identified an overlooked N_2_O sink by showing the presence of active N_2_O-consuming microorganisms in oxygenated surface seawater.

Many microorganisms participate in the marine sulfur and iron cycles via dissimilatory metabolism (Sievert et al., 2007; Melton et al., 2014). S-cycling bacteria and archaea contribute to either organic carbon consumption (via anaerobic respiration) or inorganic carbon fixation (via chemolithoautotrophy), depending on the *in situ* redox status and the available energy metabolic substrates. Jiang et al. characterized the versatile physiology and metabolic mechanisms of *Hydrogenovibrio thermophilus* strain S5, a chemolithomixotrophic hydrogen- and sulfur-oxidizing bacterium isolated from an active hydrothermal vent chimney on the Southwest Indian Ridge. The versatility of this bacterium in energy and carbon source exploitation enables its survival in the highly dynamic and harsh conditions of the deep-sea hydrothermal environments. Tang et al. investigated the microbial communities of the shallow-sea hydrothermal system off Kueishantao Island. They not only detected sulfur oxidation and carbon fixation marker gene sequences in their metagenome datasets, but also identified the signatures of many heterotrophic bacteria that harbored versatile genetic potential to adapt to the shallow-sea hydrothermal environment. Zhang et al. investigated the vertical distribution of SRB and SOXB in natural sediments of the East China Sea, a marginal sea highly impacted by riverine and anthropogenic activities. Qiao et al. investigated the mud deposit bacterial communities of the eastern China marginal seas including the East China Sea and they also quantified the *dsrB* gene abundance attributed to SRB. The work by Ihara et al. showed the successional dynamics of bacterial communities in marine sediments launched on land by earthquake-induced tsunami and identified campylobacterial SOXB as pivotal microbes during community and functional shift. This work also found the involvement of zetaproteobacterial and betaproteobacterial FeOB in sediment bacterial community succession, verifying the prevalence of FeOB in sedimentary environments of the global coastal seas (McBeth et al., 2011; Laufer et al., 2017). Chiu et al. identified two new pelagic zetaproteobacterial FeOB species from seawater of the Chesapeake Bay oxic-anoxic transition zone and—based on *in silico* genome sequence analysis—inferred their strategies for adaptation to planktonic and putative particle-associated living in aquatic environments, thereby supporting a previous finding that coastal seawater may commonly harbor biofilm-forming and biocorrosion-causing zetaproteobacterial FeOB (Dang et al., 2011). FeOB have been hypothesized as pioneer species in the initiation of carbon steel biocorrosion in marine environments, while SRB may play more important roles in biocorrosion once the biocorroding microbial communities grow into thick biofilms (Dang et al., 2011). Li et al., indeed, showed the dominance of SRB in the rust microbial communities that formed from long-term steel incubations in coastal waters.

## Regulation of Microbial Responses to Environmental Gradients and Variations

The microbial responses to environmental gradients and variations are usually highly regulated. Nawaz et al. showed the importance of small regulatory RNAs in the adaptation to deep-sea conditions in *Shewanella piezotolerans* WP3, an iron-reducing bacterium with identified piezotolerance and psychrotolerance. Furthermore, the work by Zeng et al. showed a novel molecular mechanism of *Pseudoalteromonas* sp. SM9913, a biofilm-forming marine bacterium, in adaptation to heat stress.

Coevolution of the Earth and its microbiota dictate the capability of individual microorganisms and their communities to respond to environmental changes. Although the mechanisms and processes employed by microbes to respond to environmental changes are highly diverse and complex, established biological and ecological principles are followed and can thus be decoded as suggested by the studies in this Research Topic. The general lack of available representative microbes in culture as well as experimental model systems to simulate environmental gradients presents an ongoing challenge to gaining deeper understanding of the processes, mechanisms and functions in changing marine ecosystems (Lage and Bondoso, 2012; Thøgersen et al., 2018). Continuing advancement of experimental techniques and protocols, such as those with high sampling frequency and sufficient replicates, long-term surveys, deep sequencing, systematic analyses and modeling will eventually help to reveal the mysteries of the microbial world in aquatic systems.

## Author Contributions

All authors listed have made a substantial, direct and intellectual contribution to the work, and approved it for publication.

### Conflict of Interest Statement

The authors declare that the research was conducted in the absence of any commercial or financial relationships that could be construed as a potential conflict of interest.
